# “Examining the link between tooth agenesis and papillary thyroid cancer: is there a risk factor?” Observational study

**DOI:** 10.1186/s40510-024-00511-9

**Published:** 2024-03-25

**Authors:** Željana Matošić, Luka Šimunović, Tomislav Jukić, Roko Granić, Senka Meštrović

**Affiliations:** 1 School of Dental Medicine, University of Zagreb, Zagreb, Croatia; 2 Department of Orthodontics, School of Dental Medicine, University of Zagreb, Zagreb, Croatia; 3 Department of Oncology and Nuclear Medicine, Sestre Milosrdnice University Hospital Center, 10000 Zagreb, Croatia; 4 School of Medicine, University of Zagreb, 10000 Zagreb, Croatia; 5 Department of Orthodontics, School of Dental Medicine, University of Zagreb, Zagreb, Croatia

**Keywords:** Hypodontia, Tooth agenesis, Microdontia, Palatally displaced canine, Carcinogenesis, Papillary thyroid cancer

## Abstract

**Background:**

Mutations in one or multiple genes can lead to hypodontia and its characteristic features. Numerous studies have shown a strong genetic influence on the occurrence of hypodontia, and identified several genes, including AXIN2, EDA, FGF3, FGFR2, FGFR10, WNT10A, MSX1, and PAX9, that are directly associated with dental agenesis and carcinogenesis. The objective of this study was to investigate the occurrence and pattern of tooth agenesis, microdontia, and palatally displaced canine (PDC) in women diagnosed with papillary thyroid cancer (PTC), compared to a control group of women without any malignancy or thyroid disease.

**Materials and methods:**

This case–control study was carried at the Department of Orthodontics, School of Dental Medicine University of Zagreb, and Department of Oncology and Nuclear Medicine Sestre Milosrdnice University Hospital Centre. The study involved a clinical examination and evaluation of dental status, panoramic X-ray analysis, and assessment of medical and family history of 116 female patients aged 20–40 with PTC, as well as 424 females in the control group who were of similar age.

**Results:**

The prevalence of hypodontia, microdontia, and PDC was statistically higher in women with PTC than in the control group. The prevalence rate of hypodontia was 11.3% in the experimental group and 3.5% in the control group. The experimental group showed a higher occurrence of missing upper lateral incisors, lower left central incisors, and all the third molars (except the upper left) compared to the control group. Women with PTC showed the prevalence of PDC significantly higher than the control group (3.5%, 0.7%, *p* = 0.002). The probability of hypodontia as a clinical finding increases 2.6 times, and microdontia occurs 7.7 times more frequently in women with PTC.

**Conclusion:**

Our study suggests a possible link between odontogenesis and PTC. The absence of permanent teeth may increase the likelihood of PTC in women. Leveraging the age-7 orthopantomogram to identify women at high risk for PTC within a critical early detection window could significantly improve oral health outcomes and PTC prognosis through proactive interventions.

## Background

Tooth agenesis can be caused by a range of different genetic and phenotypic factors. When defective genes interact with each other or act independently, they can lead to a specific phenotype and result in hypodontia. In recent decades, significant research has been conducted to identify the specific gene loci responsible for the most common developmental abnormalities in humans [1].

Advanced molecular genetic techniques like gene mapping have revealed links between specific gene mutations and tooth agenesis. Evidence points to hypodontia, microdontia, and palatally displaced canine (PDC) exhibiting significant and intimate genetic association. These congenital abnormalities are linked to multiple mutations in genes associated with transcription factors and growth factors that play a role in dental development [2–8].

A growing body of evidence suggests significant genetic influence on the prevalence of hypodontia including AXIN2, EDA, FGF3, FGFR2, FGFR10, WNT10A, MSX1, and PAX9, association with dental agenesis and the development of cancer [9–15].

An area of research of particular significance examines the connection between the development of cancer and the absence of teeth.

Bond et al. conducted a study on DNA samples from ovarian cancer patients to determine if there was a genetic connection between ovarian cancers and hypodontia. They identified several genes (AXIN2, MSX1, PAX9, WNT10A, EDA, BARX, and BRCA1) that may play a role in both hypodontia and cancer development. The research by Bond and colleagues demonstrated that only half of the patients with both conditions had a shared genetic cause, which significantly reduced the previously estimated risk of ovarian cancer in females with asymptomatic hypodontia [16].

Two investigations conducted by Fekonja et al. and Chalothorn et al. have found a statistically significant increase in the occurrence of congenital tooth agenesis in female patients with ovarian cancer, as compared to a control group of individuals without a diagnosis of malignancy [17, 18].

Williams et al. conducted a genome-wide association study and found a correlation between genes linked to colorectal cancer and genes associated with tooth agenesis [19]. Lammi et al. [20] identified the molecular connection between colorectal cancer and a mutation in the AXIN2 gene. Kuchler et al. demonstrated the relationship between the genes AXIN2, FGF3, FGFR10, and FGFR2 and tooth agenesis. They also reported breast and prostate cancer as the most prevalent types of cancer in their study group [21]. In 2016, Yin et al. [22] conducted research indicating that hypodontia can serve as a predictive factor for the development of colorectal, lung, breast, and ovarian cancer.

Multiple studies indicate that the canonical Wnt signaling pathway and the AXIN2 gene, which acts as a critical negative regulator to preserve the stability of β-catenin, are significant factors in cell growth, cancer development, tumor progression, and tooth formation [23–25].

The severe form of nonsyndromic hypodontia is often associated with symptoms commonly found in diseases that affect ectodermal tissues, such as issues with sweat glands, reduced saliva production, and inadequate hair or nail growth. In addition, this severe hypodontia is frequently observed in more than 120 distinct syndromes, most frequently in X-linked ectodermal dysplasia and less so in the autosomal recessive and dominant forms of hypohidrotic ectodermal dysplasia. Syndromic hypodontia is seen in patients with specific syndromes like Down, Ehlers-Danlos (Type VII), Rieger (Type I), and Witkop syndrome. Therefore, before creating a treatment plan, it is essential to rule out any related medical conditions and seek appropriate medical consultation [26].

Thyroid cancer is the most prevalent malignant disease of the women's hormone system with a significantly increasing incidence and one of the fastest growing in the USA compared to other cancers in the last 20 years. Papillary thyroid cancer (PTC) accounts for 85% of all thyroid cancers [27, 28].

Liu et al. indicated that the AXIN2 polymorphism has a significant relationship with PTC [29].

Lv and Xue's findings suggest proteins that cause rapid progression of PTC by inhibiting Wnt/-catenin and activating Axin2 [30].

A review of available literature reveals a lack of research on the potential association between hypodontia and PTC.

The objective of our study is to investigate the following issues affecting women diagnosed with PTC: (1) the prevalence of tooth agenesis, (2) the quantity of missing teeth, (3) the specific teeth that are most frequently absent, (4) whether tooth agenesis manifests unilaterally or bilaterally, (5) whether tooth agenesis is more prevalent in the maxilla or mandible, (6) the correlation between microdontia and hypodontia, and (7) the association between PDC and hypodontia.

## Methods

### Study design and sample

This case–control study was carried out at the Department of Orthodontics, School of Dental Medicine University of Zagreb, and Department of Oncology and Nuclear Medicine Sestre Milosrdnice University Hospital Centre. The approval was gained by both institutions Ethics Research Committee of the School of Dental Medicine University of Zagreb (IRB number: 05-PA-30-IV-1/2022) and the Ethics Research Committee of the Sestre Milosrdnice University Hospital Centre (IRB number: 251-29-1 J-21-01-12).

### Sample size calculation

The necessary sample size was calculated to be 98, an odds ratio of 4, a power of 0.8, and a statistical significance level of alpha less than 0.05.

### Study sample

The experimental group consisted of female patients of the Department of Oncology and Nuclear Medicine, Referral Center of the Ministry of Health for Thyroid Diseases, Sestre Milosrdnice University Hospital Center who were diagnosed with PTC aged 20 to 40.

Exclusion factors for the test group were: (1) the diagnosis of another malignant disease, (2) craniofacial and other syndromes connected with orofacial structures, (3) clefts, (4) the presence of prosthetic replacements and (5) inability to determine the reason for tooth loss.

### Informed consent

All participants have provided the Informed Consent. The research was conducted following all valid and applicable guidelines whose goal is to ensure the proper implementation of procedures and the safety of persons participating in the research, including Basic Good Clinical Practices, the Declaration of Helsinki, Act on Health Care of the Republic of Croatia (Official Gazette 100/18) and the Act on the protection of the rights of patients of the Republic of Croatia (Official Gazette 169/04, 37/08).

### Criteria

The inclusion criteria for the control group were:

(1) Age 20–40, (2) presence of panoramic radiograph regardless of the recording date. (3) Thyroid ultrasound with no detected nodules, or other abnormalities.

Exclusion criteria were (1) diagnosed malignant, autoimmune, or endocrine disease, (2) craniofacial syndromes, (3) clefts, (4) a history of childhood irradiation or chemotherapy, (5) a positive family history of any malignant or autoimmune disease, (6) the presence of prosthetic replacements and (7) inability to determine the reason for tooth loss.

**Fig. 1:**
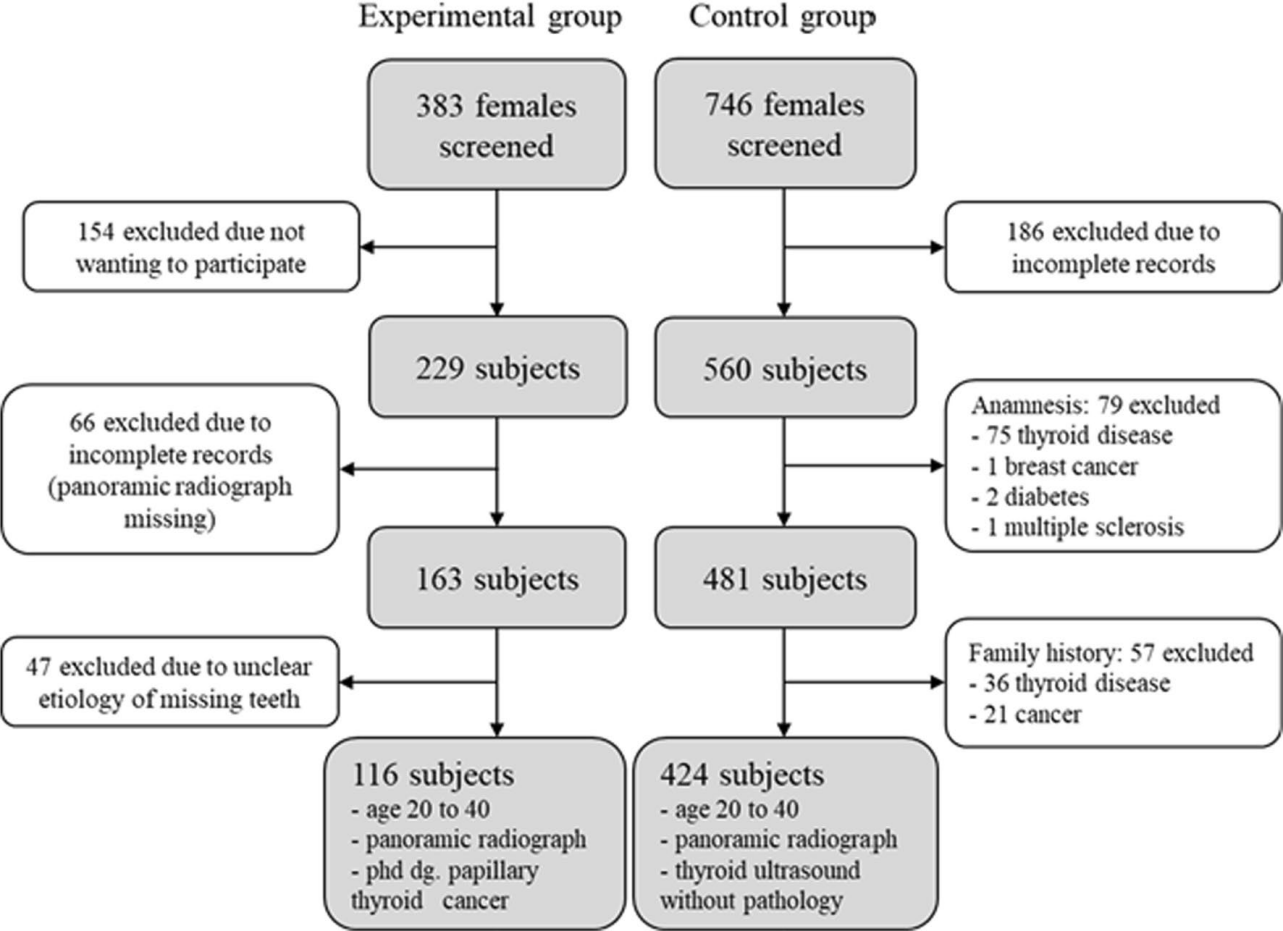
Flow diagram of subject selection

The study comprised of clinical examination and evaluation of patient dental status, panoramic X-ray analysis, medical history, and family medical history (Fig. 1).

### Data collection and variables

The research utilized the following data:

The dental examination and panoramic X-ray revealed:

1. Hypodontia of permanent teeth including third molars,
2. Microdontia of permanent teeth,
3. Impaction of upper canines.

A peg-shaped tooth was diagnosed when the incisal mesiodistal width at the crown is smaller than that of the cervical width [31]. The study employed the FDI World Dental Federation notation as its naming convention [32].

### Statistical analysis

We analyzed data using the Statistica program (TIBCO® Statistica™ version 14.0.0.15.). We assessed data distribution normality using Shapiro–Wilk and asymmetry tests. The difference in the prevalence of hypodontia between the examined and control groups was determined by the chi-square test. When the application of the approximation method was inadequate, Fisher's exact test was used for categorical data. We perform a binary logistic regression analysis to determine the impact of nonsyndromic tooth agenesis, microdontia, and PDC on PTC. Phi and Cramer V were used for the determination of the association between microdontia of upper lateral incisors and hypodontia and PDC. We use the Phi coefficient for analyzing the association between two binary variables in a 2 × 2 contingency table, where it ranges from − 1 to + 1 indicating the strength and direction of the association. Cramer’s V is appropriate for categorical variables with more than two levels in larger contingency tables, providing a value between 0 and 1 to indicate the strength of association regardless of table size. A *p* value of 0.05 or less was considered statistically significant.

## Results

The study included 116 females who had been diagnosed with PTC (the experimental group) and 424 females in the control group, all aged between 20 and 40 years. The median age of participants in the experimental group was 30, with an interquartile range (IQR) of 26–36, while in the control group, the median age was 31, with an IQR of 24–35. The prevalence of no syndromic tooth agenesis in both groups is presented in Table 1. The difference between the two groups was found to be statistically significant (*p* < 0.001).

**Table 1:** Prevalence of hypodontia, microdontia, and PDC, in the experimental (*E*) and the control (*C*) group

	*E* (%)	*C* (%)	*p*-value
Hypodontia	38.26	18.63	< .001
Hypodontia without 3rd molars	11.30	3.54	0.002
Microdontia	10.40	0.90	< .001
Microdontia without 3rd molars	7	0.90	< .001
Palatally displaced canines	3.50	0.70	0.002

**Fig. 2:**
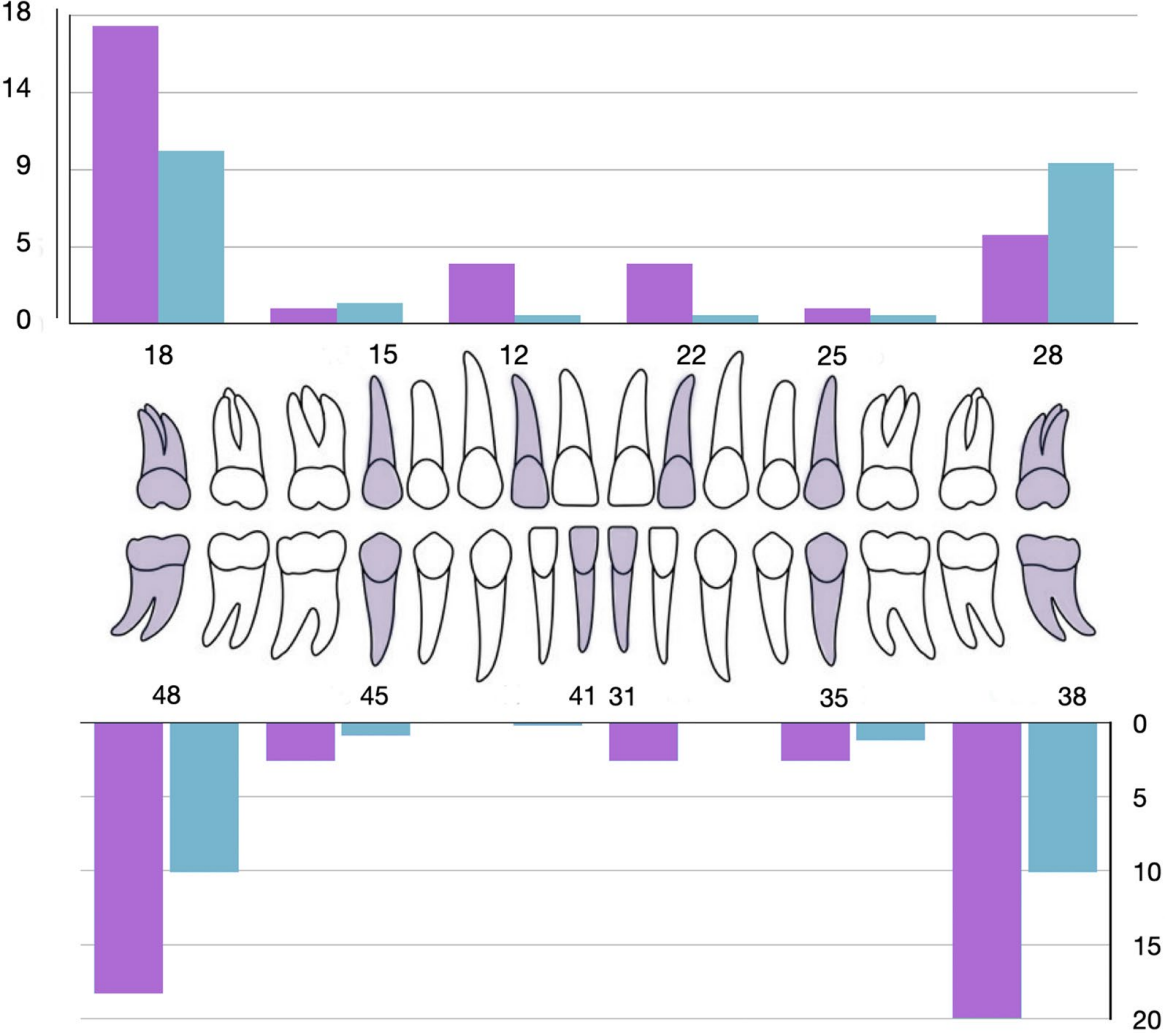
Distribution of hypodontia in the experimental (purple) and the control group (green)

The experimental group had a significantly larger percentage of hypodontia, including and excluding 3rd molars, compared to the control group. The details of the hypodontia pattern are found in Table 2 and Fig. 2. Additionally, Table 3 displays the distribution of microdontia and PDC.

**Table 2:** Prevalence (%) of hypodontia with a comparison between the experimental (*E*) and the control (*C*) group

	18			15			12			22			25			28		
	*E*	*C*	*p* value	*E*	*C*	*p* value	*E*	*C*	*p* value	*E*	*C*	*p* value	*E*	*C*	*p* value	*E*	*C*	*p* value
Bilateral	4.30%	7.80%	0.202	0%	0.20%	0.602	3.50%	0.50%	**0.006**									
Unilateral	13%	2.40%	**0.001**	0.90%	0.90%	0.942	–	–		–	–		0.90%	0.20%	0.322	0.90%	1.70%	0.535
Unilateral	3.50%	2.10%	0.401	0.90%	0.50%	0.611	0%	0.20%	0.602	2.60%	0%	**0.001**	0.90%	0.70%	0.858	5.20%	2.10%	0.074
Bilateral	14.80%	8%	**0.028**	1.70%	0.50%	0.16	–	–										
	48			45			41			31			35			38		

Bold text signifies statistical significance

**Table 3:** Prevalence of microdontia and PDC in the experimental (*E*) and the control (*C*) group

	*E* (%)	*C* (%)	*p* value
Microdontia 12	4.3	0.7	**0.015**
Microdontia 22	5.2	0.5	**< .001**
Microdontia 15	1.7	0.0	0.063
Microdontia 25	1.7	0.0	0.063
Microdontia 18	2.6	0.0	**0.009**
Microdontia 28	2.6	0.0	**0.009**
PDC 13	0.9	0.2	0.322
PDC 23	3.5	0.7	**0.02**

Bold text signifies statistical significance

In the experimental group, the upper lateral incisors were the most impacted teeth, followed by the lower 2nd premolars and lower left incisors. In contrast, the control group primarily experienced impacts on their 2nd premolars.

The occurrence of missing teeth was more common in the lower jaw (mandible) with a frequency of 55.55%, compared to the upper jaw (maxilla) with a frequency of 44.44% in the overall count. In the experimental group, bilateral absence of teeth was more frequently observed than in the control group for upper lateral incisors (3.5% vs. 0.5%, *p* = 0.006) and lower third molars (14.8% vs. 8%, *p* = 0.028) (Table 4).

**Table 4:** Distribution of hypodontia according to presence unilateral (UniLat) and bilateral (BiLat) hypodontia in the maxillary and mandibular arch

Hypodontia	*E*	*C*	*p* value	Hypodontia	*E*	*C*	*p* value
UniLat 12	–	–		UniLat 31	2.60%	0%	**0.001**
UniLat 22	–	–		UniLat 41	0%	0.20%	0.602
UniLat 15	0.9%	0.9%	0.942	UniLat 35	0.90%	0.70%	0.858
UniLat 25	0.9%	0.2%	0.322	UniLat 45	0.90%	0.50%	0.611
UniLat 18	13.0%	2.4%	**0.001**	UniLat 38	5.20%	2.10%	0.074
UniLat 28	0.9%	1.7%	0.535	UniLat 48	3.50%	2.10%	0.401
BiLat 12–22	3.50%	0.50%	**0.006**	BiLat 31–41	–	–	
BiLat 15–25	0%	0.20%	0.602	BiLat 35–45	1.70%	0.50%	0.16
BiLat 18–28	4.30%	7.80%	0.202	BiLat 38–48	14.80%	8%	**0.028**

Bold text signifies statistical significance

Statistically significant variations were observed between groups in terms of the presence of additional dental abnormalities, microdontia, and tooth impaction.

The experimental group displayed higher values for both microdontia and PDC, indicating a significant association between microdontia of upper lateral incisors and hypodontia and PDC (Table 5). We conducted a binary logistic regression analysis to determine the impact of hypodontia and microdontia (with or without M3) as well as PDC on the likelihood of participants having PTC (Table 6). The model accounted for 12.4% (Nagelkerke *R*2) of the variance and accurately classified 80.3% of the sample. Among the five predictor variables, only hypodontia (*p* < 0.001) and microdontia (*p* = 0.003) were statistically significant at 5% level. Hypodontia increased the probability of being in the PTC group by 2.6 times, while microdontia increased it by 7.7 times (with a wide confidence interval).

**Table 5:** Significant association (*p* < 0.001) presented as Phi coefficient between microdontia of upper lateral incisors and hypodontia and PDC

	Hypodontia 31	Hypodontia 25	PDC 13	PDC 23
Microdontia 12	0.197	–	0.244	0.121
Microdontia 22	0.197	0.197	0.244	0.12

**Table 6:** Possible risk factors for PTC

	*B*	Lower and upper CI	*p* value
Hypodontia	2.613	1.567–4.357	**< .001**
Hypodontia excluding M3	1.542	0.633–3.753	.340
Microdontia	7.724	2.012–29.657	**.003**
Microdontia excluding M3	2.654	0.534–13.186	.233
Palatally displaced canines	3.5	0.635–19.289	.150

Bold text signifies statistical significance

*M3, third molar; CI, confidence interval

## Discussion

Agenesis of permanent teeth has shown a major increase over the twentieth century, however the observed time span is too short and too limited for such a statement [33, 34]. Another study, analyzing tooth agenesis trends from 2001 to 2021, indicated that the USA is a leader in tooth agenesis research. The study, which reviewed 2287 journal articles, pointed out that multidisciplinary management is the preferred treatment for dental agenesis. Research on gene mutations related to tooth agenesis continues to be a significant area of interest, with a potential future research direction being the exploration of the relationship between tooth agenesis and cancer [35]. Genetic studies have demonstrated that some malignant diseases such as colorectal cancer, lung or breast cancer suggest a direct connection between carcinogenesis and tooth agenesis [17–20, 36, 37]. Recent studies have highlighted a potential connection between tooth agenesis and carcinogenesis, suggesting a shared genetic basis between these conditions. The genes involved in odontogenesis, the process of tooth development, have been found in tumor tissues or cells, indicating an overlap in the genetic pathways of both processes. These include genes such as PAX9, MSX1, AXIN2, EDA, EDAR, WNT10A, and others involved in WNT signaling [37]. This genetic interconnection suggests that tooth agenesis could be an early marker for cancer predisposition, as variants in these genes might serve as early indicators or therapeutic targets for cancer [36, 37]. Additionally, studies have shown a correlation between the genetic determinants of nonsyndromic tooth agenesis and the development of neoplasms in adulthood [38]. This emerging research direction not only contributes to a deeper understanding of the development of dental anomalies and cancer but also opens new avenues for early diagnosis and treatment strategies, emphasizing the significance of genetic research in both fields [36, 39].

The transcription factor, muscle segment homeobox 1 (MSX1) is crucial in the process of abnormal proliferation and tumorigenesis because it controls both the activation and suppression of other genes [40–42].

MSX1 encodes critical genes that control a variety of morphogenetic processes, including such as the development of teeth, and bones, and the growth and proliferation of various cell types [43–47]. As a transcription factor, MSX1 is involved in multiple epithelial-mesenchymal interactions during vertebrate embryogenesis and demonstrates pleiotropic effects across several tissues [48]. In humans, MSX1 variations are associated with dental anomalies like tooth agenesis and orofacial clefting, as well as other conditions like nail dysplasia [48]. Notably, the location of mutations within the MSX1 gene influences the resulting phenotypes. Variants affecting the homeodomain (HD) of MSX1 typically cause tooth agenesis, with or without additional phenotypes. In contrast, mutations outside the HD are mostly linked with non-syndromic orofacial clefts (nsOFC) [48].

MSX1's role in abnormal cell proliferation and tumorigenesis is also significant [49, 50]. Epigenetic silencing of MSX1 through DNA methylation can lead to different phenotypes or even increase the risk for tumor growth [50]. This implies a complex interplay between genetic and epigenetic factors in the manifestation of MSX1-related conditions.

In recent literature the influence of MSX1 in tooth agenesis is controversial.

Vastardis et al. propose that MSX1 is a crucial gene for the regular growth of teeth. Their study indicated that a mutation in the MSX1 gene causes hypodontia [51]. Research by Mostowska et al. [52] also supports the idea of the significant role of MSX1 in hypodontia. Their findings showed for the first time that the MSX1 gene's novel cT671C mutation may be the etiological variant significantly involved in familial cases of hypodontia that only affect the second premolars and third molars. The findings of our study support the Vastardis and Mostowska statement as we identified a weak positive but significant association between agenesis of the first upper right premolar and the third upper left molar.

However, some researchers have found no connection between MSX1 gene mutation, and tooth agenesis [53–55].

The findings of our study indicate a potential connection between the two phenotypes, odontogenesis, and PTC. The incidence of permanent tooth agenesis in females ranges from 6 to 10% excluding third molars and it is 1.37 times higher than in males [21, 56].

In our study, there was a significant increase in the prevalence rate of hypodontia 11.3% (excluding M3) in female patients with PTC over the prevalence obtained in the control group (3.5%). Our experimental group showed a significantly higher prevalence of dental agenesis of upper lateral incisors, lower left central incisors, and all the third molars except the upper left over the prevalence of mentioned teeth in the control group (Table 2). Also, we reported a significantly higher prevalence of bilateral agenesis of maxillary second incisors in the experimental than in the control group. The unilateral absence of the maxillary second incisor was not found. Lupinetti's study suggested the unilaterally absent lateral incisors are less frequent than the bilaterally absent ones. In addition, 62.5% of patients with maxillary second incisor agenesis had a tooth that was a peg on the opposite side [57].

Our study reports that hypodontia as a clinical finding in these subjects increases the probability 2.6 times that the subject has a PTC in comparison to healthy control.

These results support previous similar studies of hypodontia as a risk marker in women with cancer but are the first ones connecting PTC and hypodontia. Chalothorn et al. found a statistically significant difference in the prevalence of hypodontia in patients with ovarian cancer (20%) compared to a healthy control group (3%) [18]. A direct association between the occurrence of cancer and hypodontia was reported by Fekonja et al. They discovered a prevalence of tooth agenesis of 19.2% in female patients with ovarian cancer compared to 6.7% in females in the control group [17].

The majority of prior epidemiologic research on hypodontia has purposely excluded third molars due to the difficulty of putting together a properly aged sample for taking accurate third molar data. In his study, Peck suggested third molar status may offer helpful proof for improved comprehension of phenotypic patterns and specific genetic processes [58]. Henriksson et al. suggest that from the Middle Ages to the present, there has been no increase in the prevalence of third molar agenesis [59]. The findings of the mentioned studies encouraged us to include third-molar data in our research. Prevalence of third molar agenesis in women with PTC was found 38.3% compared to 18.6% in the control group which supports our idea of the importance of including third molar agenesis data in the study.

The PDC seems to be an anomaly in development with genetic causes but precise etiology is not known up to date [60–63]. In the literature, Pax9 polymorphism is the most often mentioned as a responsible factor for palatal impaction of upper canines [64–71]. Devi et al. [68], in particular, aimed to evaluate the association between PAX9 polymorphisms and palatally impacted canines. In this study, single nucleotide polymorphisms (SNPs) rs12532 of MSX1 and rs2073247 of PAX9 were genotyped in a sample of fifty individuals with palatally impacted maxillary canines and fifty age and gender-matched controls. The study found a statistically significant association between these SNPs and palatal impaction of maxillary canines. Specifically, the presence of the AG/CT genotypes of these genes in an individual was linked to a significantly increased risk for palatal impaction. These findings suggest a positive association between rs12532 and rs2073247 polymorphisms of MSX1 and PAX9 genes and the palatal impaction of maxillary canines, pointing toward a genetic basis for this dental anomaly.

Despite previous research indicating that dominant major genes are responsible for the etiology of PDC, the involvement of additional minor genes, as well as possible environmental or epigenetic influences on gene transcription, could modify the phenotype [60, 66, 72–76].

Studies have found that dental abnormalities like hypodontia and impaction of maxillary canines are caused by specific genetic mechanisms [58, 60, 74, 77]. Pirinen et al. reported that individuals with PDC have a 4.5 times higher prevalence of dental agenesis compared to the general population [61].

Our research indicates that PDC (palatally displaced canine) was significantly more prevalent in women with PTC (papillary thyroid carcinoma) compared to the control group (3.5% vs 0.7%). Lagana et al. [78] proposed that the only condition directly linked to a displaced maxillary canine is the absence of maxillary lateral incisors. Our findings demonstrate a moderate correlation between impaction of the upper canines and microdontia (abnormally small size) of the mentioned teeth. Specifically, impaction of the upper left canine is associated with agenesis (absence) of the upper left and right lateral incisors (Phi = 0.14).

Sacerdoti and Baccetti [79] found that missing upper lateral incisors were significantly associated with unilateral PDC, while missing third molars were significantly associated with bilateral PDC. However, they did not find any association between missing upper lateral incisors and missing third molars. The difference in sample sizes between studies may explain this discrepancy. Our research reveals a significant difference in the prevalence of small upper lateral incisors in women with PTC compared to women without cancer (4.3% vs 0.7%; 5.2% vs 0.5%). To our knowledge, no previous study has investigated the link between small teeth and cancer.

This investigation revealed a robust correlation between microdontia of the upper lateral incisor and agenesis of the lower left incisor, as well as microdontia of the upper lateral incisor and PDC. These findings provide evidence in favor of Pecks' notion of three genetically coexisting dental defects, as proposed in reference [58].

The study had several limitations, including a small sample size of women with PTC and dental anomalies, and the use of panoramic radiography as the main diagnostic method. Increasing the sample size would be advantageous in obtaining a more accurate estimate of the prevalence and patterns of tooth agenesis in this population, resulting in a narrower confidence interval. Additionally, the study was observational in nature, which means it could only identify associations and not establish causality. Conducting more studies with larger sample sizes, as well as incorporating familial and genetic analyses, would be essential in furthering our understanding of this topic. Regarding the Phi coefficients discussed earlier, there is no universally fixed threshold for clinical significance for Phi coefficients. Values above 0.5 are often considered strong associations and may be of clinical relevance, especially when they have practical implications for decision-making or patient care. However, the context of the study, field-specific standards, and the impact on patient outcomes should always be taken into account when determining clinical significance. Moreover, while radiographic examinations, such as panoramic radiographs, are valuable tools for assessing tooth size and morphology, they provide a two-dimensional representation of a three-dimensional structure. This limitation can result in potential inaccuracies in measurements and may not capture subtle variations in tooth size accurately. Additionally, radiographic assessments do not always account for the functional or clinical significance of microdontia, which can vary among individuals. Therefore, the diagnosis of microdontia based solely on radiographic findings should be complemented by clinical evaluation (such as conducted in this research) to ensure a comprehensive understanding of the condition and its potential impact on oral health and function.

## Conclusion

The results of this study provide evidence for a potential association between tooth agenesis and the occurrence of PTC. The absence of permanent teeth may serve as an early indication of the potential onset of PTC, as it increases the likelihood by a factor of 2.6. Given that the recommended age for the initial orthopantomographic X-ray is 7 years, it could be straightforward to identify females who are at a heightened risk of developing PTC.

## Data Availability

The datasets used and/or analyzed during the current study are available fromthe corresponding author on reasonable request.

## Abbreviations

PDC: Palatally displaced canine
PTC: Papillary thyroid cancer
